# Supplementary material for: A deep learning sex-specific body composition ageing biomarker using dual-energy X-ray absorptiometry scan
Source: Commun Med (Lond). 2025 May 13;5:171. doi: 10.1038/s43856-025-00850-6 (PMC12075649; doi:10.1038/s43856-025-00850-6)

**Supplementary Methods**

**Supplementary Method 1. VAT and lean mass percentage calculation**

The VAT values were derived using field ID 23288 and the lean mass using field ID 23280. The VAT and lean mass were normalized using total mass (field ID 23282).

**Supplementary Method 2. Disease field ID and ICD-10 Codes**

T2DM: Category 1712, "E10”, “E11"

MACE: Category 1712, "G45", "I21", "I22", "I23", "I24", "I25", "I63", "I64"

ASCVD: Category 1712, "I20", "I21", "I22", "I23", "I24", "I25", "I63", "I65", "I66", "I70",

"I71", "I72", "I73", "I74", "I77", "I78", "I79"

Hypertension: Category 1712, "I10", "I11", "I12", "I13", "I15"

**Supplementary Method 3.** UK Biobank DXA imaging protocol

DXA scans were measured by GE-Lunar iDXA from 3 centres.

Scans at the whole body, lumbar spine and hip sites are analysed by the radiographer at, or soon after, acquisition to generate all numerical measures of bone mass and body composition. Images (whole body, hip, knee, AP lumbar spine and lateral thoraco-lumbar spine) were provided without further processing.

Information is from [https://biobank.ndph.ox.ac.uk/ukb/refer.cgi?id=502.](https://biobank.ndph.ox.ac.uk/ukb/refer.cgi?id=502)

**Supplementary Method 4. BCBA model development, process and the computational environment**

- Model development: Pytorch DenseNet 121 as backbone and added an external layer for regression task. Average pooling was used. The raw age label was used as the final target.

(Normalised age was also tried but get similar results as raw age.)

- Image Processing: The two input images were initially centre cropped and then normalised. The two processed image were then concatenated into one image as the input.
- Environment: Python 3.8, Pytorch 1.12, sklean 1.30, numpy 1.24 and pandas 2.0.3.

**Supplementary Tables**

**Supplementary Table 1**: ResNet-50, VGG-16, Vision Transformer and Voting Ensembled model comparing with DenseNet121 on testing set (combine male and female).

| Model | MSE | R score |
| --- | --- | --- |
| CNN | 24.087 | 0.744 |
| VGG-16 | 18.411 | 0.809 |
| ResNet-50 | 18.016 | 0.815 |
| Vision Transformer | 17.552 | 0.824 |
| Voting Ensemble model | 14.453 | 0.865 |
| DenseNet121 | 14.012 | 0.866 |

**Supplementary Table 2**: Female MAPE and MSE per age group

|  |  |  | Female | |  |  |
| --- | --- | --- | --- | --- | --- | --- |
| Met  rics | Group | Overall | 40-49 | 50-59 | 60-69 | 70-79 |
| MA  PE | Normal  Reference Test | 0.047,  (0.045, 0.05) | 0.077, (0.06,  0.094) | 0.052,  (0.048,  0.057) | 0.04, (0.037,  0.043) | 0.045, (0.04,  0.05) |
|  | Pre-existing Disease | 0.051, (0.05,  0.052) | 0.106,  (0.092,  0.119) | 0.062,  (0.059,  0.065) | 0.045,  (0.044,  0.047) | 0.049,  (0.047,  0.051) |
|  | Post-DXA  Disease | 0.048,  (0.045, 0.05) | 0.105,  (0.088,  0.121) | 0.063,  (0.056,  0.069) | 0.041,  (0.038,  0.044) | 0.042,  (0.038,  0.046) |
| MS  E | Normal  Reference Test | 13.168,  (11.875,  14.46) | 21.735,  (12.562,  30.908) | 13.544,  (11.287,  15.801) | 11.04,  (9.361,  12.719) | 15.638,  (12.575,  18.701) |
|  | Pre-existing Disease | 17.006,  (16.28,  17.732) | 34.301,  (26.699,  41.903) | 18.388,  (16.728,  20.047) | 13.747,  (12.932,  14.561) | 20.357,  (18.734,  21.979) |
|  | Post-DXA  Disease | 15.171,  (13.691,  16.652) | 31.635,  (22.81,  40.459) | 19.138,  (15.395,  22.88) | 12.166,  (10.334,  13.997) | 15.42,  (12.491,  18.349) |

**Supplementary Table 3**: Male MAPE and MSE per age group

|  |  |  | Female | |  |  |
| --- | --- | --- | --- | --- | --- | --- |
| Met  rics | Group | Overall | 40-49 | 50-59 | 60-69 | 70-79 |
| MA  PE | Normal  Reference Test | 0.052,  (0.049,  0.055) | 0.093,  (0.073,  0.114) | 0.058,  (0.052,  0.063) | 0.044,  (0.041,  0.048) | 0.047,  (0.042,  0.053) |
|  | Pre-existing Disease | 0.052,  (0.051,  0.053) | 0.104,  (0.091,  0.117) | 0.067,  (0.064, 0.07) | 0.046,  (0.045,  0.048) | 0.049,  (0.047, 0.05) |
|  | Post-DXA  Disease | 0.051,  (0.049,  0.053) | 0.098,  (0.076,  0.119) | 0.06, (0.055,  0.066) | 0.046,  (0.044,  0.049) | 0.048,  (0.045,  0.052) |
| MS  E | Normal  Reference Test | 15.793,  (14.137,  17.45) | 30.224,  (19.354,  41.095) | 16.112,  (13.04,  19.185) | 12.728,  (10.7,  14.757) | 17.786,  (14.037,  21.535) |
|  | Pre-existing Disease | 18.395,  (17.733,  19.056) | 38.819,  (30.572,  47.065) | 21.529,  (19.922,  23.136) | 14.764,  (13.989,  15.538) | 20.562,  (19.255,  21.87) |
|  | Post-DXA  Disease | 17.639,  (16.32,  18.958) | 32.779,  (19.662,  45.896) | 17.936,  (15.025,  20.846) | 14.874,  (13.305,  16.443) | 20.788,  (17.93,  23.647) |

**Supplementary Table 4**: R scores for different healthy groups

|  | Female |  | Male |  |
| --- | --- | --- | --- | --- |
| Normal Reference  Test |  | 0.872 |  | 0.858 |
| Hypernormal |  | 0.823 |  | 0.835 |
| Suboptimal |  | 0.815 |  | 0.840 |
| Pre-existing Disease |  | 0.815 |  | 0.803 |
| Post-DXA Disease |  | 0.837 |  |  |

**Supplementary Table 5**: C-index of model 1-3 comparison for ASCVD and MACE

|  |  | ASCVD | MACE |
| --- | --- | --- | --- |
| Female | Model 1 | 0.656 (0.611, 0.700) | 0.645 (0.607, 0.678) |
|  | Model 2 | 0.631 (0.607, 6.654) | 0.610 (0.579, 0.640) |
|  | Model 3 [our model] | **0.679** (0.643, 0.710) | **0.678** (0.666, 0.691) |
|  | Model 1+3 | 0.684 (0.672, 0.695) | 0.689 (0.645, 0.722) |
|  | Model 2+3 | 0.632 (0.599, 0.668) | 0.683 (0.639, 0.714) |
| Male | Model 1 | 0.632 (0.598, 0.669) | 0.639 (0.615, 0.663) |
|  | Model 2 | 0.604 (0.582, 0.626) | 0.635 (0.611, 0.659) |
|  | Model 3 [our model] | **0.643** (0.601, 0.684) | **0.641** (0.620, 0.669) |
|  | Model 1+3 | 0.703 (0.635, 0.741) | 0.686 (0.654, 0.700) |
|  | Model 2+3 | 0.640 (0.599, 0.692) | 0.649 (0.612, 0.681) |

Supplementary Figures

**Supplementary Figure 1:** Best performed model training, validation, and test loss

0

5

10

15

20

25

30

35

40

45

1

4

7

10

13

16

19

22

25

28

31

34

37

40

43

46

49

52

55

58

61

64

MSE

DenNet

121

Loss

Plot

train loss

val loss

test loss

**Supplementary Figure 2:** Scatter plots of BCBA-CA vs. CA for female. The X axis is CA while the Y axis is BA- CA.


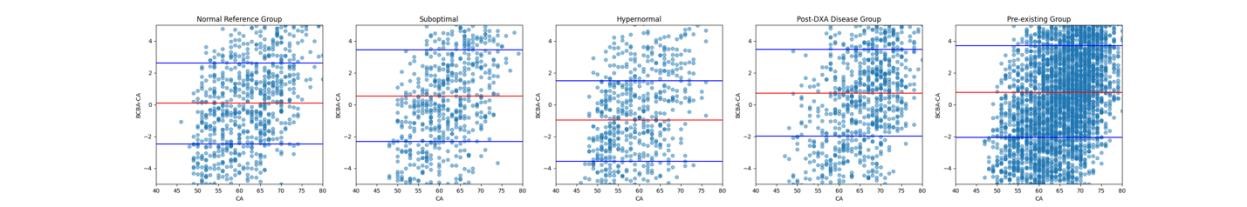


**Supplementary Figure 3:** Scatter plots of BCBA-CA vs. CA for males. The X axis is CA while the Y axis is BA- CA.


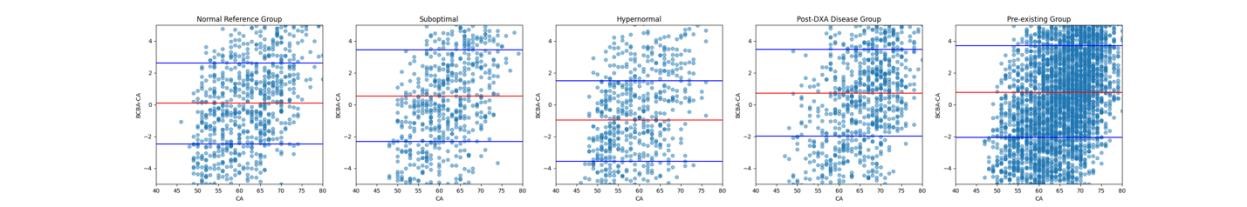

Supplement: Supplementary file 2 — Supplementray Material [file 43856_2025_850_MOESM2_ESM.docx]
